# Investigating the Influence of Processing Conditions on Dissolution and Physical Stability of Solid Dispersions with Fenofibrate and Mesoporous Silica

**DOI:** 10.3390/pharmaceutics16050575

**Published:** 2024-04-24

**Authors:** Ana Baumgartner, Nina Dobaj, Odon Planinšek

**Affiliations:** University of Ljubljana, Faculty of Pharmacy, Aškerčeva 7, 1000 Ljubljana, Sloveniaodon.planinsek@ffa.uni-lj.si (O.P.)

**Keywords:** mesoporous silica, amorphous solid dispersion, solvent evaporation method, fenofibrate dissolution, physical stability

## Abstract

The study aimed to enhance the solubility of the poorly water-soluble drug, fenofibrate, by loading it onto mesoporous silica, forming amorphous solid dispersions. Solid dispersions with 30% fenofibrate were prepared using the solvent evaporation method with three solvents (ethyl acetate, acetone, and isopropanol) at different temperatures (40 °C, boiling point temperature). Various characteristics, including solid-state properties, particle morphology, and drug release, were evaluated by different methods and compared to a pure drug and a physical mixture of fenofibrate and silica. Results revealed that higher solvent temperatures facilitated complete amorphization and rapid drug release, with solvent choice having a lesser impact. The optimal conditions for preparation were identified as ethyl acetate at boiling point temperature. Solid dispersions with different fenofibrate amounts (20%, 25%, 35%) were prepared under these conditions. All formulations were fully amorphous, and their dissolution profiles were comparable to the formulation with 30% fenofibrate. Stability assessments after 8 weeks at 40 °C and 75% relative humidity indicated that formulations prepared with ethyl acetate and at 40 °C were physically stable. Interestingly, some formulations showed improved dissolution profiles compared to initial tests. In conclusion, mesoporous silica-based solid dispersions effectively improved fenofibrate dissolution and demonstrated good physical stability if prepared under appropriate conditions.

## 1. Introduction

Good aqueous solubility is a key parameter to ensure drug absorption in the gastrointestinal tract after oral application, which is crucial for achieving therapeutic drug concentrations and thus the desired therapeutic outcome. However, the aqueous solubility of many newly discovered active pharmaceutical ingredients (APIs) is very low, as they are mostly classified as Class II or IV according to the Biopharmaceutical Classification System (BCS) [1,2]. Since oral application is most desired by both the pharmaceutical industry and patients, many chemical and physical approaches have been introduced and explored to improve poor water solubility [3]. Among them, the formulation of amorphous solid dispersions is one of the most promising strategies. When a substance is transformed from a crystalline to an amorphous state, it loses the ordered structure of a crystal lattice and thus gains higher molecular mobility and higher free energy states, leading to improved solubility and dissolution rates [4]. However, the lack of an ordered structure is also the reason why amorphous materials tend to recrystallize and lose the above-mentioned advantages [5].

**Figure 1:**
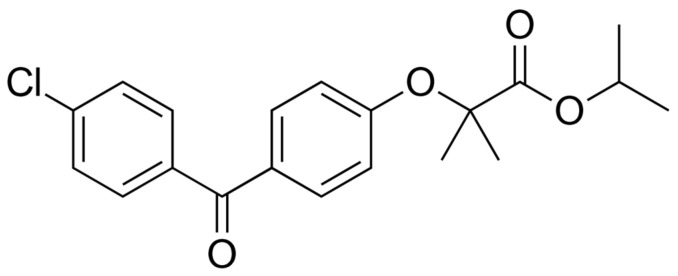
Fenofibrate.

The transformation of the crystalline state into an amorphous state can be of use even for improving the solubility of the most challenging compounds [6]. These fall into Class IIb according to the Developability Classification System (DCS), which is based on BCS, but it divides BCS Class II into dissolution rate-limited (DCS Class IIa) and solubility-limited (DCS Class IIb) compounds. Unlike for Class IIa compounds, the overall absorption of Class IIb compounds cannot be improved simply by increasing the dissolution rate but rather by increasing intrinsic solubility. The latter is considered a greater challenge, which is possible to overcome only by altering the chemical structure or the solid state of compounds [6]. Fenofibrate (FF; Figure 1), a cholesterol-lowering agent belonging to the BCS Class II and DCS Class IIb, is a lipophilic substance with an aqueous solubility of less than 0.1 mg/mL, which was selected as a model substance for our study [6,7,8]. It was already reported as a model compound to have dissolution and solubility improved by amorphization, but its low glass transition temperature (<−20 °C) makes it particularly challenging to maintain the amorphous state and thus the physical stability of the formulations [2]. A solution to this problem could be the incorporation of FF in mesoporous materials, as the confinement of molecules in narrow pores can prevent the formation of crystallization nuclei and thus crystal growth [3,9].

Indeed, mesoporous materials, especially mesoporous silicon dioxide, i.e., silica, are gaining increasing research interest as carriers for amorphous solid dispersions (SDs). If an otherwise poorly water-soluble drug is adsorbed onto such a material, a larger surface area and the amorphous state can contribute to improved dissolution properties. In addition, the process of adsorption leads to a reduction of the free energy in the system, which is another factor that can improve physical stability [5,10]. So far, the applicability of mesoporous silica in the preparation of amorphous solid dispersions with improved dissolution properties has been demonstrated for many poorly water-soluble drugs, such as carvedilol [11,12], itraconazole [13], indomethacin [14], carbamazepine, celecoxib, griseofulvin, ritonavir [15], and many others. Apart from their application as carriers in SDs, mesoporous silica materials, especially nanoparticles, are also intensively researched as nanoplatforms for cancer immunotherapy, gene therapy, targeted drug delivery, etc. However, these platforms are mostly based on tailor-made silica particles with an ordered and well-defined pore structure, which are not yet approved for use in pharmaceutical products. For more information about these applications, the reader is referred to the recent extensive reviews [16,17,18].

There are already many types of mesoporous silica available on the market and used in pharmaceutical products as glidants or anti-tacking agents, all of which have a non-ordered pore structure. The materials can differ in many physical properties, e.g., particle size, average pore size, specific surface area (SSA), pore connectivity/geometry, etc. These properties can significantly affect the extent of drug loading and the drug release profile. It has been suggested that small particles with relatively large mesopores are better suited for higher drug loading as well as faster drug release [14,19]. Syloid^®^ 244 FP, a commercially available mesoporous silica used in our study, fulfills these requirements well, as its average particle size is below 3.5 µm, its average pore size is 16 nm, and its SSA is approximately 300 m^2^/g [10,20].

Several drug loading methods have been proposed for loading active ingredients onto mesoporous silica, which can be divided into solvent-based and solvent-free methods [3]. In addition to the particle properties already mentioned, the drug loading method can also influence the amount of drug loaded, its distribution, its physical state, and its release [14,21]. In solvent-based methods, organic solvents are generally used to load the drug into a mesoporous carrier. The rotary evaporation method is one of the most commonly used techniques on a laboratory scale and can also be used on an industrial scale. First, the mesoporous carrier is added to the drug solution, and then the solvent is removed in a rotary evaporator under vacuum. An important advantage of this method is that as the solvent evaporates, the concentration gradient between the mesoporous particles and the surrounding drug solution slowly increases, driving the drug into the pores [14,22]. Lai et al. [22] loaded ibuprofen into different types of mesoporous silica materials by rotary evaporation, starting with different initial drug concentrations and different ratios of drug to silica. They showed that faster drug release can be achieved by a lower drug/carrier ratio and a lower initial drug concentration, while the physical state of the drug is mainly affected only by drug/silica ratio. They also emphasized that the influence of solvent on drug loading and dissolution performance could be significant and needs to be investigated. Different drug/silica ratios and their effect on SD properties have also been the subject of research in some other studies [11,23,24,25], but at least to our knowledge, the effect of solvent on the degree of amorphicity, drug release, and stability has not been investigated yet. Furthermore, the effect of solvent temperature has also not been explored, although it could influence SD characteristics by altering the solubility of the drug in a given solvent and thus the nature of drug loading inside the pores.

The aim of our study was to prepare amorphous SDs with FF and mesoporous silica by the rotary evaporation method and to investigate the influence of different conditions (solvent type, solvent temperature, FF/silica ratio) on the properties of SDs. Changes in the solid state were evaluated by X-ray powder diffraction (XRPD), differential scanning calorimetry (DSC), and attenuated total reflectance Fourier transform-infrared (ATR FTIR) spectroscopy. These results were correlated with drug release experiments in a discriminatory medium. In addition, we aimed to investigate the physical stability of the prepared formulations after storage at 40 °C and 75% relative humidity (RH) and to find out whether different preparation conditions affect them.

## 2. Materials and Methods

Mesoporous silica (Syloid 244 FP, referred to in the text as Syloid) was obtained from Grace Davison, Grace GmbH & Co. KG (Worms, Germany). FF was obtained from Biosynth^®^, Carbosynth (Berkshire, UK). All other materials used in the study were of reagent grade. Water was purified by reverse osmosis.

### 2.1. Preparation of SDs

SDs with FF and Syloid were prepared by the solvent evaporation method in a rotary evaporator (IKA RV 05, Staufen, Germany). Three different solvents (ethyl acetate, acetone, and isopropanol) were used for the preparation, which differ in their FF dissolution capacity. FF (2.4 g) was dissolved in 100 mL of solvent, followed by the addition of Syloid (5.6 g). This corresponded to a theoretical content of 30% FF in SD. The suspensions obtained were evaporated in a rotary evaporator at 50 rpm under two different conditions: (1) 40 °C, reduced pressure, and (2) boiling point temperature (77 °C for ethyl acetate, 82 °C for isopropanol, 56 °C for acetone), normal atmospheric pressure [23]. This process took up to 30 min. After the solvent was not visible in the flask anymore, the pressure was lowered to <10 mbar for 30 min to ensure complete removal of the solvent. With ethyl acetate at boiling point temperature conditions, SDs with different other FF contents (20, 25, and 35%) were prepared. The choice of FF content range was based on preliminary experiments, which showed that FF remains partially in the crystalline state at ratios above 40%, so it would not be feasible to increase the FF ratio above this threshold. A physical mixture (PM) was prepared by mixing silica and FF for approximately 5 min in a 3D motion mixer (Inversina, Bioengineering, Wald, Switzerland). The conditions for each prepared formulation and its theoretical content are listed in Table 1. The annotation “high” in the formulation labels indicates the preparation at the boiling point temperature, whereas “low” stands for the preparation at 40 °C.

### 2.2. Characterization

#### 2.2.1. DSC

DSC measurements were performed for each freshly prepared SD to assess the solid-state form of FF in the formulations. Examinations were performed using the DSC1 STARe system (Mettler Toledo, Columbus, OH, USA). The samples (5–10 mg) were heated in an aluminum pan with a perforated lid from 0 °C to 110 °C at a rate of 20 °C/min and under nitrogen gas flow of 50 mL/min. An empty aluminum pan was used as a reference. Output data were evaluated by the STARe V9.30 software. DSC of pure FF and a 30% physical mixture were recorded for reference.

#### 2.2.2. XRPD

XRPD diffractograms were used as a second method for solid-state assessment of FF inside the SDs. The measurements were performed by a PANalytical PW3040/60 X’Pert PRO diffractometer (Malvern Panalytical, Worcestershire, UK), with CuKα1 radiation, λ = 1.5406 Å, using the continuous scanning mode in the 2θ range from 5 to 40° and a step of 0.033° per 100 s. Spectra of pure crystalline FF, Syloid, and a 30% physical mixture were recorded as well.

#### 2.2.3. ATR FTIR

ATR FTIR analysis was used to check for potential interactions between FF and Syloid. The spectra were obtained by the Nicolet Nexus FTIR Spectrometer (Nicolet Instruments, Madison, WI, USA). A diamond ATR accessory (DuraSample IR Technologies, Danbury, CT, USA) was employed for these experiments. The measurement range of the IR spectra was from 500 to 4000 cm^−1^, the resolution was 2 cm^−1^, and the data were recorded as the average of 64 iterations. The data were analyzed by OMNIC 9 software (Nicolet CZ, Prague, Czech Republic). The literature data were used to assign the peaks to specific functional groups.

#### 2.2.4. Particle Size and Morphology

The particle size of FF, Syloid, PM, and SDs was measured using the laser diffraction method (Malvern Mastersizer 3000, Malvern Instruments, Worcestershire, UK) with a dry powder feeder. The parameters used were the following: feed air pressure of 2.5 bar, 0.5–6% obscuration rate, and an approximation theory setting for non-spherical particles, where the refractive index of silica (n = 1.45) was applied. Each sample was measured in triplicate to calculate the d10, d50, and d90 (volumetric parameters indicating the fraction of particles smaller than 10%, 50%, and 90% of the analyzed particles, respectively) and the standard deviation.

The morphology of FF, Syloid, and the prepared formulations was studied by scanning electron microscopy (SEM). The particles were deposited on a double-sided carbon tape (diameter 12 mm, Oxon, Oxford Instruments, Oxford, UK) and coated with a thin layer of gold before observation to increase the clarity of the images. A SEM (JSM-6060 LV, Jeol, Tokyo, Japan) with an accelerating voltage of 10 kV and a secondary detector was used. The samples were scanned with a magnification of 500×.

#### 2.2.5. Nitrogen Adsorption Studies

The SSA was determined via nitrogen adsorption isotherms (Tristar 3000, Micromeritics, GA, USA) at 77 K. Before the measurement, the samples (0.1–0.2 g) were outgassed in a vacuum oven at 45 °C overnight. The calculation of SSA was based on the multipoint Brunauer-Emmett-Teller equation (BET) in the relative pressure range of 0.05 to 0.3 [26]. The total pore volume was estimated by the t-plot method at the highest applied relative pressure [27]. The average pore radius was derived using the BJH (Barrett-Joyner-Halenda) model [28].

#### 2.2.6. Determination of FF Content

The FF content was determined using UV spectroscopy with absorbance measured at wavelength 290 nm. Precisely weighed SD samples were suspended in acetone to extract FF. After 10 min ultrasonication, the samples were filtered through 0.45 µm pore RC membrane filter to remove silica particles and sufficiently diluted prior to the absorbance measurement. The concentration of FF was determined based on previously obtained calibration curves of FF in acetone. The actual FF content in SDs was expressed as % of the theoretical content.

#### 2.2.7. Drug Release from SDs

Drug release experiments were performed in a USP II apparatus with rotating paddles (VanKel VK 7010 Tablet Dissolution Tester, VanKel Technology Group, Cary, NC, USA). Samples of SDs containing 150 mg of fenofibrate were placed into 900 mL of the selected discriminatory dissolution medium at 37.5 °C ± 1 °C (0.1 M HCl pH = 1.2, 0.01 M SDS, 0.034 M NaCl). The dissolution conditions were non-sink, because we aimed to use the amount of FF which corresponds to a therapeutic dose. At the specified time points (5, 10, 15, 30, 45, 60, 90 and 120 min), 5 mL of medium was withdrawn and filtered through a 0.45 µm pore RC membrane filter. The withdrawn medium was not replaced, but the reduction in total volume was considered in drug release calculations. The samples were further analyzed with UV spectroscopy, with absorbance measured at 290 nm. FF concentration was determined in relation to previously obtained calibration curves of FF in the dissolution medium. Drug release profiles were plotted as the cumulative percentage of released FF versus time. The experiments were performed in triplicates to calculate the average and the standard deviation for each formulation.

To assess the similarity between dissolution profiles, the similarity factor *f_2_*, proposed by Moore and Flanner, was calculated. This is a widely used and model-independent approach used to mathematically assess whether two dissolution profiles are similar [29]. It is calculated by Equation (1):

(1) $$f_{2}=50\times \log\left\{{\left[1+\frac{1}{n}\sum_{j=1}^{n}{\left|R_{j}-T_{j}\right|}^{2}\right]}^{-0.5}\times 100\right\},$$

where *n* is the number of time points at which the sample was withdrawn, *R_j_* is the percentage dissolved at time point *j* for reference formulation, and *T_j_* is the percentage dissolved at the same time point for test formulation. The value *f_2_* lies between 0 and 100, and a value larger than 50 indicates similarity, whereas a value below 50 indicates dissimilarity [30].

To determine the solubility of FF in the dissolution medium at 37 °C, an excess amount of FF was added to 20 mL of medium so that a portion of FF dissolved and a larger part remained undissolved. The suspension was heated to 37 °C in a water bath and stirred overnight. The suspension was filtered through a 0.45 µm pore RC membrane filter to obtain a clear saturated solution, which was then analyzed by UV spectroscopy with absorbance measured at wavelength 290 nm. The experiment was carried out in duplicate.

### 2.3. Physical Stability

To evaluate the physical stability of SDs, samples were stored at 40 °C and 75% RH for 8 weeks. They were evaluated at 4 weeks by the drug release experiment and at 8 weeks by DSC measurement, XRPD, and the drug release experiment (same procedures as described previously). These results were compared to results at time zero (evaluations on the day of preparation) to assess whether samples remained unchanged during the storage period.

## 3. Results and Discussion

First, six different SDs with Syloid and FF in equal proportion were successfully prepared by the rotary evaporation method in three different solvents at two different temperatures for each solvent. In the second part of the study, the best conditions were used for the preparation of three more SDs with different proportions of FF. All these samples were subjected to a stability test at 40 °C/75% RH for 8 weeks. The measured FF content in all prepared SDs ranged from 90.1% to 97.5%, indicating that the incorporation of FF into silica particles was successful.

### 3.1. Particle Size and Morphology of FF, Syloid, PM, and SD Particles

**Figure 2:**
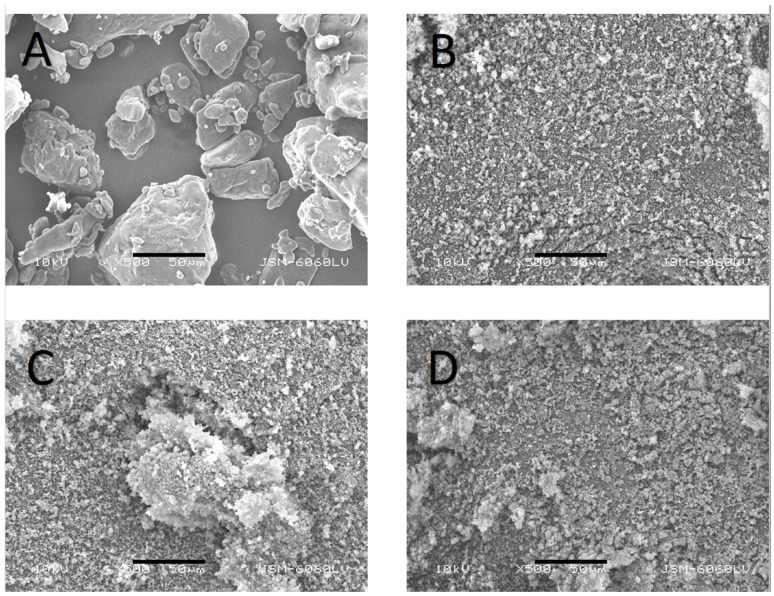
SEM images of FF (**A**), Syloid (**B**), PM-30 (**C**) and an example of a SD [EA-25 (**D**)] taken at 500× magnification. The black scale bar marks 50 µm.

The SEM images of crystalline FF, Syloid, PM-30, and an example of SD are shown in Figure 2. The morphology of the two starting substances, FF and Syloid, is seen to be very different. While FF appears in the form of smooth crystals of irregular shapes and sizes, Syloid particles are more uniform in size, and its particles are much smaller than the particles of FF. This was confirmed by particle size measurements, where the average d50 equaled 2.81 ± 0.02 µm for Syloid and 84.43 ± 2.20 µm for FF. In the physical mixture PM-30, it can be seen that the Syloid particles either stand alone or are adhered to the much larger FF particle in the middle of the image. As the SDs were not seen to differ a lot from one another, only one example is shown in Figure 2 (SD-EA-25). The particles resemble those seen in the image of Syloid, which suggests that during the rotary evaporation process, the dissolved FF enters the Syloid pores and adheres to their surface. However, some particle clusters consisting of several small particles can be distinguished, which is probably due to some FF having adsorbed to the Syloid surface, which caused the particles to agglomerate. This was also evident from the measured d50 of SD particles, which was somewhat higher for SDs (3.30 ± 0.34 µm; the given average and standard deviation were calculated from the measurements of all SDs, since the results among them were similar) than for Syloid. In addition, structures of Syloid particles adsorbed to the surface of small FF particles were seen in some samples, although this was a rare occurrence. When measuring the particle size, this phenomenon was seen in the relatively large differences in the average d90, which ranged from 18.45 ± 20.14 µm (SD-EA-20) to 232 ± 44 µm (SD-AC-low), as this is probably dependent on the number of agglomerated particles in the measured sample. This means that the samples were not completely homogenous, which is not surprising considering that rotary evaporation was used, where the drying process is not as fast and uniform as in some other solvent-based methods of SD preparation (e.g., spray-drying).

### 3.2. Influence of Solvent Type and Solvent Temperature on Physico-Chemical Properties and Dissolution

**Figure 3:**
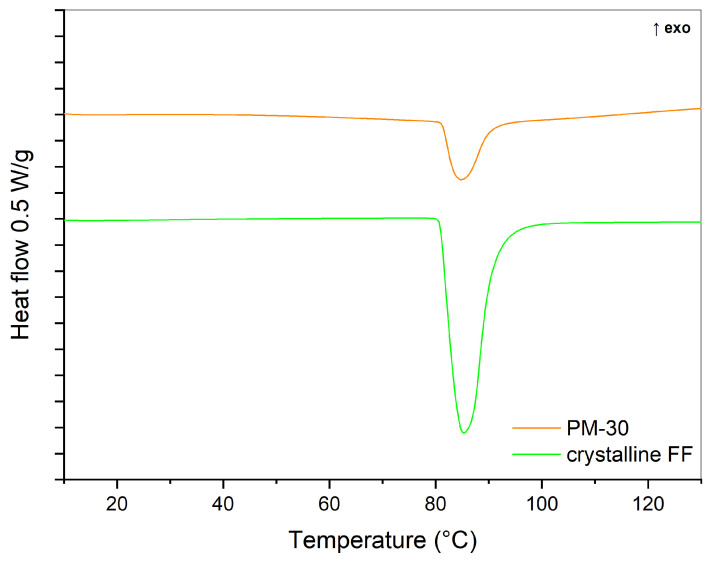
DSC thermogram of crystalline FF and 30% physical mixture (PM-30).

**Figure 4:**
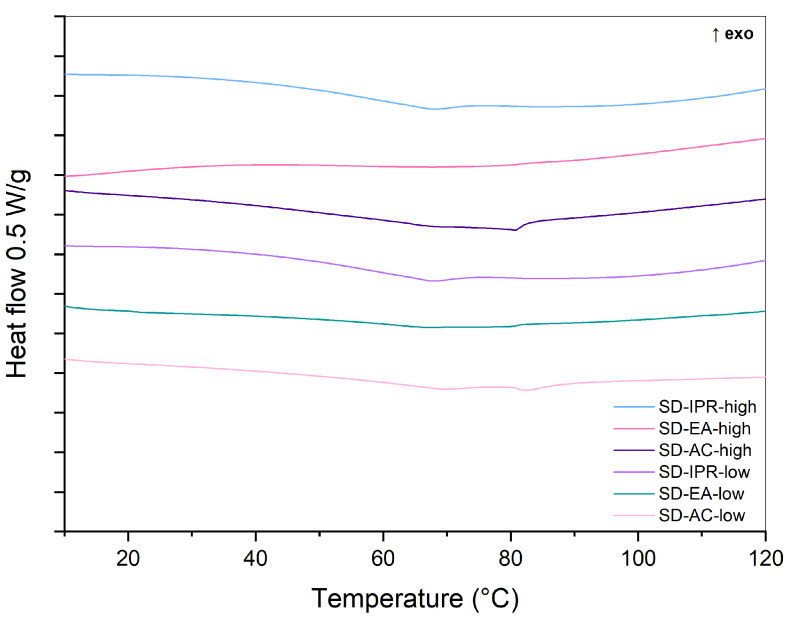
DSC curves of SDs containing 30% FF prepared under different conditions (solvent, temperature).

DSC was used to evaluate the physical state of the prepared samples. As a reference, DSC curves of pure crystalline FF and a 30% physical mixture (PM-30) were recorded (Figure 3). The melting peak of crystalline FF is seen at an onset temperature of approximately 80 °C, which is in line with published literature [31,32]. The enthalpy of fusion, calculated by integrating the endothermic peak, was 85 J/g. This endothermic peak can also be seen in the thermogram of PM-30, indicating that FF was present in crystalline form. However, its area, which correlates to the enthalpy of fusion, is smaller (21 J/g) because there is only 30% FF in the analyzed sample, and probably because part of FF is adsorbed on the silica surface. The DSC curves of the prepared SDs are shown in a separate figure (Figure 4), because the changes in heat flow were much smaller there. These results indicate that the amount of crystalline FF, if any, is much lower in SDs than in physical mixtures and that most of it is dispersed molecularly or has turned into an amorphous state. No endothermic events are observed in the SD-EA-high formulation, while small endothermic peaks are seen in all other SDs, suggesting that some FF is still present in crystalline form. While some samples only show a peak at around 80 °C, a small endothermic event with enthalpy below 2 J/g was also seen at onset temperatures between 60 and 65 °C in some samples (SD-IPR-low, SD-EA-low, SD-AC-low, SD-IPR-high). This could indicate the presence of smaller FF crystals, which formed inside the smaller pores due to spatial constrictions, while the peak at 80 °C could be a sign of FF crystallization in larger pores and on the surface of Syloid [22]. Another possible explanation is that the API is present in a disordered crystalline state within the pores, which is distinguished by a broader peak at temperatures lower than the melting point, as stated by Matsumoto et al. for solid dispersions of ethenzamide on porous crystalline cellulose [33].

**Figure 5:**
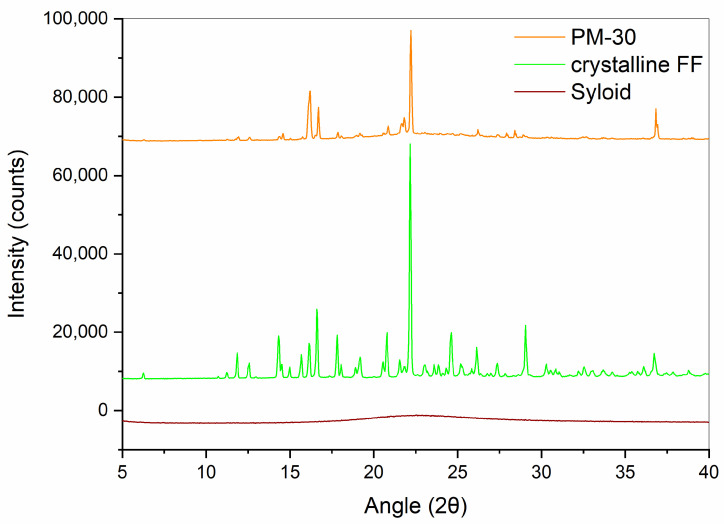
XRPD spectra of crystalline FF, PM-30 and Syloid.

**Figure 6:**
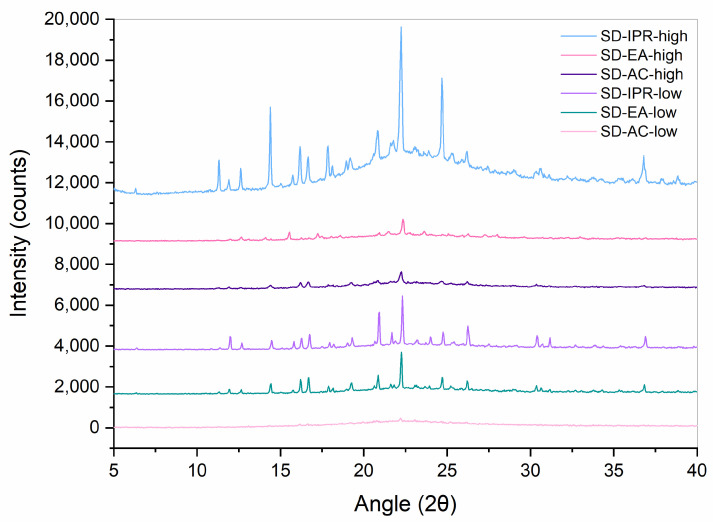
XRPD spectra of SDs containing 30% FF prepared under different conditions (solvent, temperature).

XRPD measurements were performed in order to confirm the results obtained with DSC. Diffractograms of crystalline FF, PM-30, Syloid, and SDs with 30% FF prepared under different conditions are shown in Figure 5 and Figure 6. While Syloid does not give any distinct peaks, crystalline FF shows distinct Bragg peaks at 2θ 14.3, 16.6, 17.8, 20.8, 22.2, 24.6, and 29.0, which is in line with the published literature [32,34,35]. PM-30 also shows distinct peaks at most of these values, implying that FF is present in crystalline form, as was already postulated with DSC. However, their intensity is lower, as the amount of FF in the sample is 30%. The peaks in the SDs prepared with acetone and ethyl acetate are very low, if at all (note the difference in the intensity scale in Figure 5 and Figure 6), which means that crystalline FF is almost completely absent in these formulations. This is also consistent with the DSC results, with the exception of SD-EA-high, where DSC shows no endothermic events but a small peak corresponding to crystalline FF is seen in the XRPD spectrum. In formulations prepared with isopropanol, the intensities of crystalline peaks are higher, which should indicate a greater proportion of crystalline FF [36].

**Figure 7:**
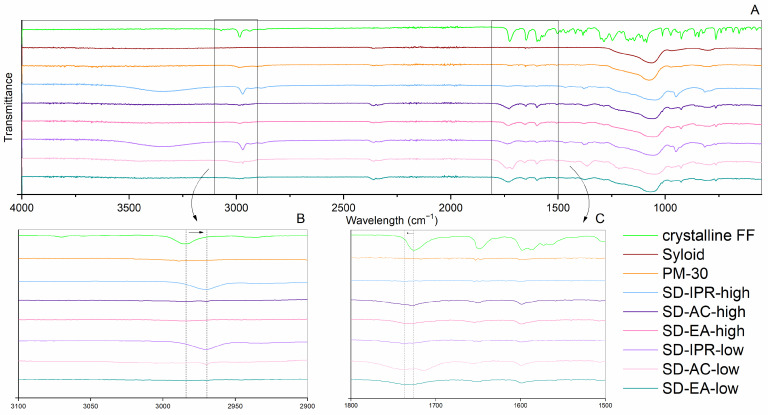
FTIR spectra of crystalline FF, Syloid, PM-30 and SDs prepared under different conditions (solvent, temperature); whole spectra (**A**) and magnifications of particular spectral regions (**B**,**C**).

To gain a deeper insight into potential interactions within the formulations, which may affect drug release and physical stability, ATR FTIR was recorded for each prepared formulation and compared to the spectra of pure crystalline FF and pure Syloid. All spectra are shown in Figure 7. The peaks that are clearly seen in the spectra of FF are at approximately 2985 cm^−1^ (C-H stretching of the isopropyl group), 1726 cm^−1^ and 1649 cm^−1^ (C=O bonds stretching), 1597 cm^−1^ and 1588 cm^−1^ (in-plane benzene ring stretch), 1247 cm^−1^ and 1146 cm^−1^ (C–O bonds stretching), and other peaks in the spectral region below 1200 cm^−1^ [32,37,38]. Syloid shows a strong intensity band at 900–1300 cm^−1^, which is characteristic of Si–O stretching [22]. In some formulations, weak bands are seen in the range of 3500–3300 cm^−1^ due to O–H stretching of the silanol group and hydrogen bonds between the silanol groups and water or solvent molecules that remained in the samples [22,39]. Otherwise, there are no new peaks in the prepared SDs, which means that no chemical bonds have been formed and that fast drug release can be expected in the presence of water. However, in some formulations, small peak shifts can be seen for the peak at 2985 cm^−1^ and 1726 cm^−1^ to 2969 cm^−1^ and 1730–1736 cm^−1^, respectively (note the dashed lines in the magnified figures within Figure 7). According to Figari et al., shifts towards lower wavenumbers in the spectral region 2900–3000 cm^−1^ could be due to either amorphization or the build-up of interactions with the silanol groups on the pore walls [38]. The peak shift from 1726 cm^−1^ to higher wavelengths, together with band broadening, is consistent with changes reported for amorphous FF [32,38]. Of the peaks around 1600 cm^−1^ associated with the in-plane benzene ring stretching, only the peak with the higher wavelength is retained in the prepared formulations (see the dashed circle in the magnified figure within Figure 7).

**Figure 8:**
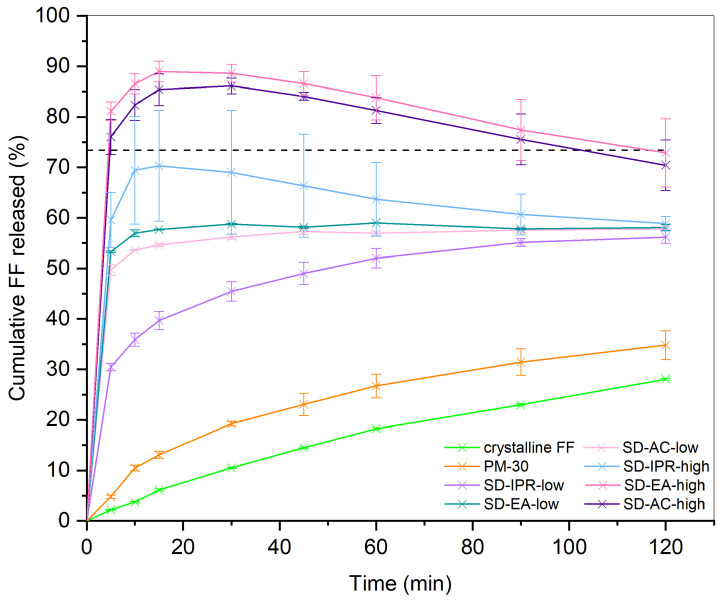
Drug release from SDs containing 30% FF prepared under different conditions (solvent, temperature). Dashed line marks the thermodynamic solubility of crystalline FF in dissolution medium, which was determined according to the method explained in Section 2.2.7.

The drug release behavior of formulations prepared at different conditions is shown in Figure 8. As a reference, dissolution of pure crystalline FF was performed, and it can be observed that less than 30% dissolved in 120 min, which corresponds to less than half of its thermodynamic solubility in this medium at 37 °C (the solubility line is marked in the graph with a black dashed line). A slight improvement in drug release can be seen in PM-30, where FF concentration after 120 min was 1.24 times higher than for pure crystalline FF. This could be due to simple particle size reduction as a result of the mixing process, which may lead to faster dissolution. However, as fenofibrate is a DCS class IIb drug, meaning that more complex approaches are needed to improve its dissolution, the increase is not very prominent. It is also possible that FF adsorbs to the Syloid surface during physical mixing, which is hydrophilic and therefore improves wettability. Improved drug release from physical mixtures of drug and silica compared to pure drug has already been reported and discussed in several studies dealing with different APIs [30,31,40].

All prepared SDs show an improved drug release profile compared to pure FF and a physical mixture with the same FF/Syloid ratio, which means that the adsorption of FF onto the porous structure is crucial for achieving fast drug release. This is because FF is largely converted from a crystalline to an amorphous state in such a system, as confirmed with DSC and XRPD, and this is associated with higher dissolution rates compared to the pure crystalline form. In addition, increased wettability and greater surface area available for dissolution, both resulting from adsorption onto the silica pores, can also lead to faster drug release. It is also important to note that no chemical bonding occurs during the process, which was confirmed by FTIR analysis.

**Figure 9:**
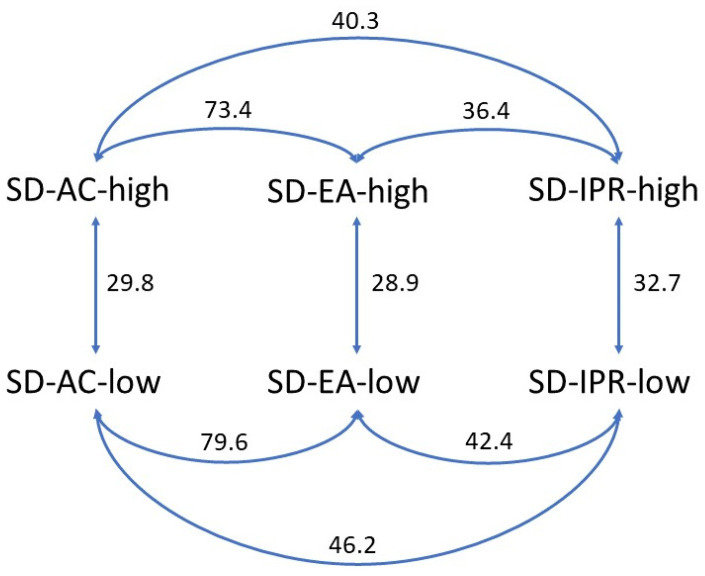
Pairwise comparison of similarity between dissolution profiles by similarity factor f_2_; a value higher than 50 indicates similarity between the profiles of two formulations, whereas a value lower than 50 indicates dissimilarity.

However, certain differences between the dissolution profiles arise due to the different preparation conditions. It is evident that the solvent temperature affects the dissolution rate as well as the extent of FF released; SDs prepared at 40 °C all show slower drug release than those prepared at higher temperatures with the same solvent, which was also predicted by the FTIR results. The difference is indicated by the calculated similarity factors *f_2_* shown in Figure 9, which are all well below 50 for pairs of dissolution profiles with the same solvent at different temperatures, indicating that these profiles are dissimilar (29.8, 28.9, and 32.7 for SDs made in acetone, ethyl acetate, and isopropanol, respectively). This observation can be explained by the fact that drug solubility depends not only on the solvent but also on the temperature of the solvent. As the solvent starts to evaporate, the API concentration gradually increases until saturation solubility is reached. When this point is exceeded, API begins to crystallize, unless it is already adsorbed on the silica surface. As drug solubility decreases at lower temperatures, the API solution reaches saturation earlier, i.e., at a lower concentration, which is why it is more likely to start precipitating in a crystalline form. In contrast, higher temperatures lead to higher solubility, and thus the concentration gradient, which promotes FF diffusion into the silica particles, can be maintained for longer.

There are also differences between the dissolution profiles of SDs prepared with different solvents, although these are not as evident as with the solvent temperature (also indicated by the *f_2_* values being closer to 50 or even higher). The solvent that gave SDs the lowest dissolution rates was isopropanol, both at 40 °C and at the boiling point temperature at normal atmospheric pressure. This can also be explained by the fact that FF is the least soluble in isopropanol among the three solvents [41]. On the other hand, the solubility of FF in ethyl acetate and acetone is very similar, as are the dissolution profiles of formulations prepared in these two solvents, according to the *f_2_* values (73.4 and 79.6 for boiling point temperature and 40 °C, respectively) [41,42]. Another factor that can influence the adsorption of the API onto the silica surface and the extent of adsorption is the polarity of the solvent and its possible interactions with the silanol groups. The measure of polarity is the dielectric constant; the higher the value, the more polar the solvent [43]. According to the literature, polar solvents compete with the API more strongly than non-polar solvents for adsorption binding sites on the silica surface, which is why the adsorption of the API onto the pores can be compromised in this case [22,44]. Ethyl acetate is the least polar of all solvents, with a dielectric constant of 6.02 (at 20 °C), while for acetone and isopropanol, these values are 20.7 and 18.3, respectively [45]. Although SDs prepared with ethyl acetate have a slightly faster drug release than those prepared with acetone, this difference is not very large, and the profiles are considered similar according to the *f_2_* value. Therefore, it seems that solvent polarity does not play an important role in our case and that there are other, more important factors affecting the drug dissolution rate.

### 3.3. Influence of FF to Syloid Ratio on Physico-Chemical Properties and Dissolution

To date, several studies have been published evaluating the effects of drug/silica ratio on SD properties (amorphicity, dissolution rate, stability, etc.). However, the optimal ratio to achieve the best results seems to be case-specific, i.e., it depends on both API and silica properties, drug loading method, etc., which is why we also wanted to investigate this issue in our case. We prepared SDs with different API amounts using ethyl acetate at boiling point temperature, as these conditions were the best according to the evaluation of physical state and dissolution. As mentioned above, preliminary experiments have shown that SDs with 40% FF or more always result in partially crystalline API, leading to slower drug release and poor physical stability, which is not the aim of formulating SDs.

Nitrogen adsorption studies were performed on the samples prepared with the best conditions and a varying amount of FF. All samples exhibited a type IV adsorption isotherm, characterized by a hysteresis loop indicating a mesoporous material (isotherms not shown). The SSA, pore volume, and average pore diameter of the starting material (Syloid), and SDs are given in Table 2. Pore volume and SSA generally decrease with the increasing API load, presumably due to the mesopores being occupied by the FF molecules, as was reported previously [11,21]. The decrease, however, is not linear, which is likely due to the precipitation of small FF crystals on the Syloid particle surface or to the crystals forming inside the mesoporous network, which occlude the pores and cause a reduction in pore volume [1]. On the other hand, only small changes in the average pore diameter can be seen between the SDs and the starting material, which has been reported before [11,46], but needs further investigation to elucidate the likely causes.

**Figure 10:**
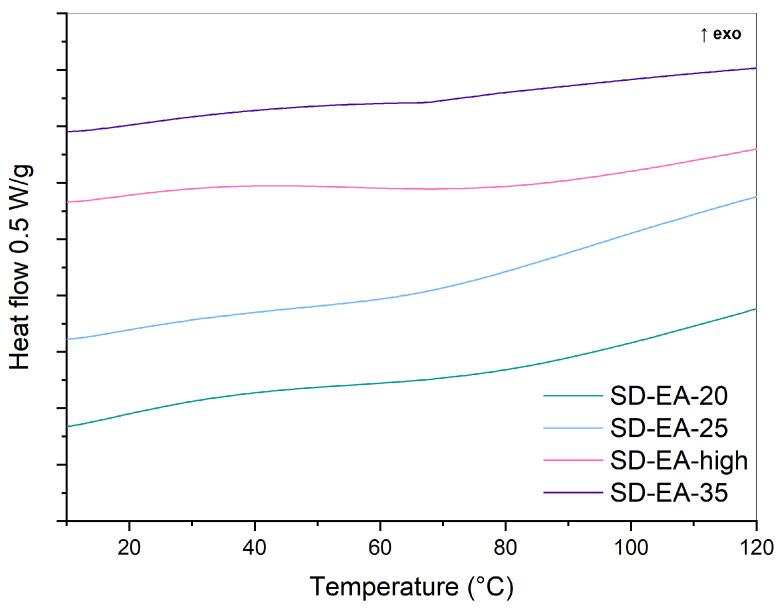
DSC curves of SDs prepared with different amounts of FF in ethyl acetate at boiling point.

Figure 10 shows DSC curves for SDs with different amounts of FF. No endothermic events are seen in any of the samples, indicating that no crystalline FF was present in the prepared formulations. This shows that under the best conditions of preparation, i.e., in ethyl acetate at boiling point temperature, amorphous SDs with even more than 30% FF can be prepared. This finding shows that our approach is promising, since the previously published literature on amorphous SDs with silica and FF does not report amorphous SDs with such high amounts of FF. For example, Jia et al. prepared SDs with FF, Sylysia^®^ 350, and Eudragit L by rotary evaporation from ethanol and proved the absence of crystalline FF at 17% FF content, while Water et al. detected signs of crystallinity at 17% FF content in SDs with silica core shell material, prepared by the microwave irradiation method [23,31].

**Figure 11:**
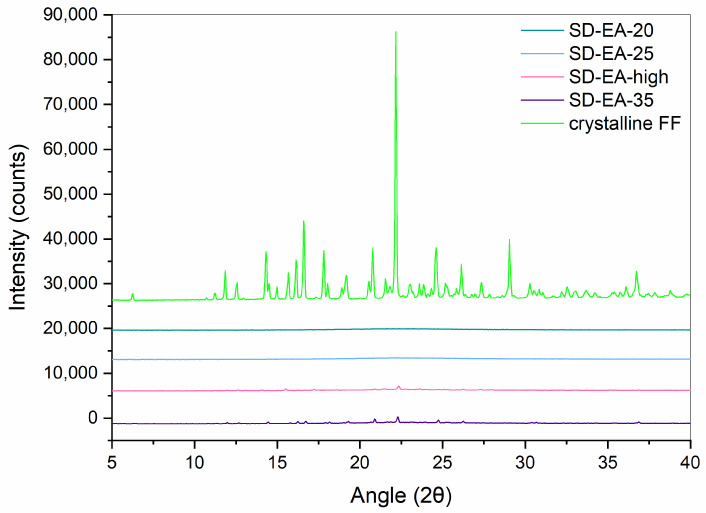
XRPD curves of SDs prepared with different amounts of FF in ethyl acetate at boiling point temperature.

XRPD spectra of crystalline FF and SDs with different FF amounts prepared in ethyl acetate at boiling point temperature are seen in Figure 11. In SD-EA-20 and SD-EA-25, no distinct peaks can be seen, only a halo between 10 and 30°, which suggests that FF is completely amorphous in these formulations. On the other hand, the XRPD spectra of SD-EA-high and SD-EA-35 show small peaks that coincide with peaks of crystalline FF, indicating traces of crystalline FF in these formulations. However, these results are in conflict with the DSC results, from which it seems that there should be no crystalline FF in any of the samples. The reason for this contradiction could be simply the difference in sensitivity between the two methods, or it could happen that the melting peak in the DSC curve cannot be distinguished due to the rising baseline. Either way, the discrepancy cannot be fully explained.

**Figure 12:**
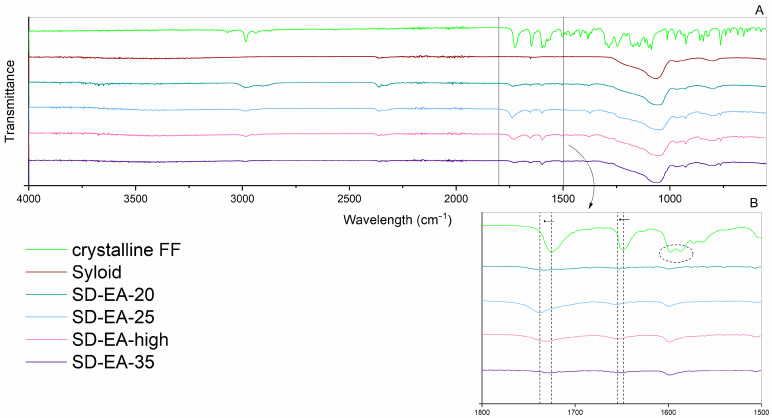
FTIR spectra of crystalline FF, Syloid and SDs prepared with different amounts of FF in ethyl acetate at boiling point temperature; whole spectra (**A**) and a magnification of a particular spectral region (**B**).

The FTIR spectra of formulations with different amounts of FF are shown in Figure 12. As with the other prepared SDs, there are no new peaks in the spectra, meaning the absence of new chemical bonds. Interestingly, the peak at 2985 cm^−1^ does not show a shift to lower wavelengths in any of the formulations, in contrast to what was previously observed. However, more peak shifts can be observed in the other spectral regions; in addition to the previously explained peak shift from 1726 cm^−1^ to higher wavelengths, the peak at 1648 cm^−1^ is shifted towards 1654 cm^−1^, which is again in line with the spectrum of amorphous FF [32,38]. For the peaks around 1600 cm^−1^, which are assigned to the in-plane benzene ring stretching, only the one with the higher wavelength is retained, as previously described.

**Figure 13:**
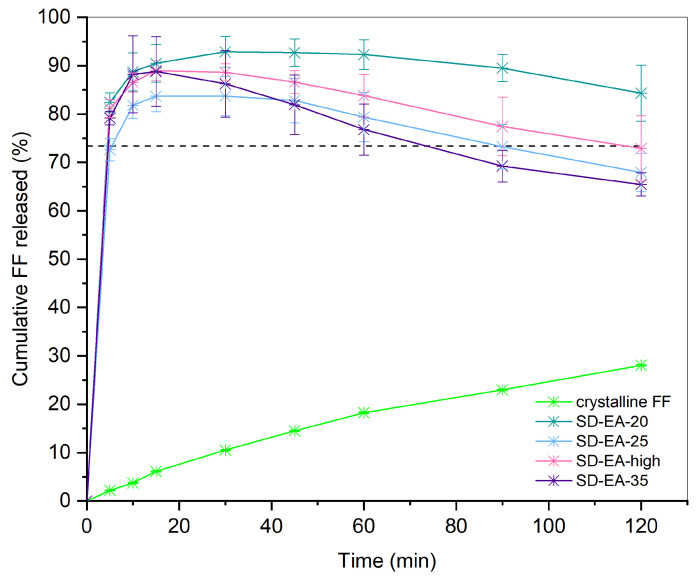
Drug release from of SDs prepared with different amounts of FF in ethyl acetate at boiling point temperature. Dashed line marks the thermodynamic solubility of crystalline FF in the dissolution medium, which was determined according to the method explained in Section 2.2.7.

Drug release from SDs prepared in EA at boiling points with different FF ratios is shown in Figure 13. Again, all SDs performed significantly better than crystalline FF, which was expected based on the results from DSC and XRPD. Furthermore, the differences between the formulations are relatively small, especially in the first few minutes, when supersaturation was reached at all FF ratios (all dissolution profiles intersect the solubility line). Compared to pure crystalline FF, where less than 5% of the dose was dissolved in the first 10 min, all SDs showed more than 80% release at the same time (89%, 82%, 87%, and 88% for SD-EA-20, SD-EA-25, SD-EA-high, and SD-EA-35, respectively). However, after the initial supersaturated state, a decrease in dissolved API is observed in all formulations, indicating that precipitation occurs. It appears that higher amounts of FF in SD lead to a higher degree of precipitation, as the FF concentration in SD-EA-high and SD-EA-35 was the lowest at the end.

It is worth mentioning that in none of the prepared SDs did the drug release reach 100% at any time point (see Figure 8 and Figure 13), meaning that it was incomplete. This has been previously described in the literature, and it is believed that there is a dynamic adsorption equilibrium between the drug adsorbed to silica and the free drug in a dissolution medium [47,48]. However, while in our case, drug release was higher at lower drug loadings, Le et al. [47] found higher drug release at high drug loadings when they loaded a different type of commercially available mesoporous silica (Syloid^®^ XDP 3050) with felodipine and furosemide. Another possible explanation could be that some drug molecules are too strongly bound to the silica surface to be displaced by water molecules.

### 3.4. Physical Stability

**Figure 14:**
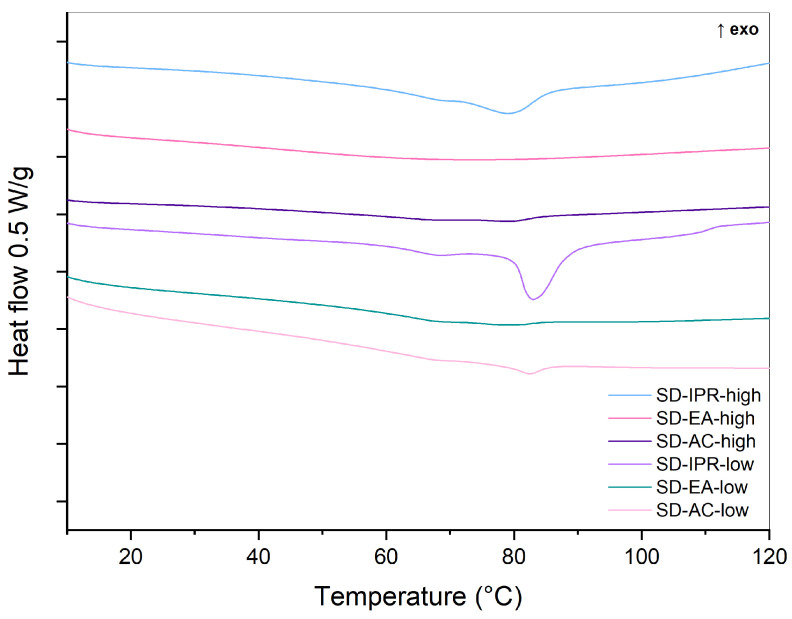
DSC curves of SDs containing 30% FF prepared under different conditions (solvent, temperature) after 8 weeks of storage at 40 °C.

**Figure 15:**
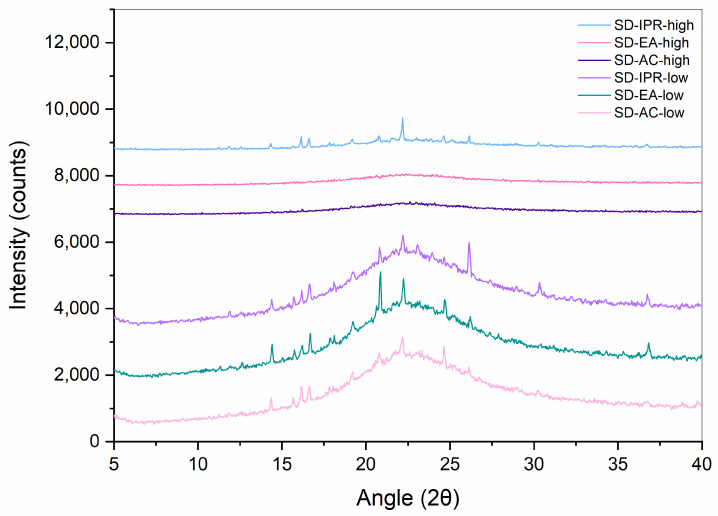
XRPD spectra of SDs containing 30% FF prepared under different conditions (solvent, temperature) after 8 weeks of storage at 40 °C.

**Figure 16:**
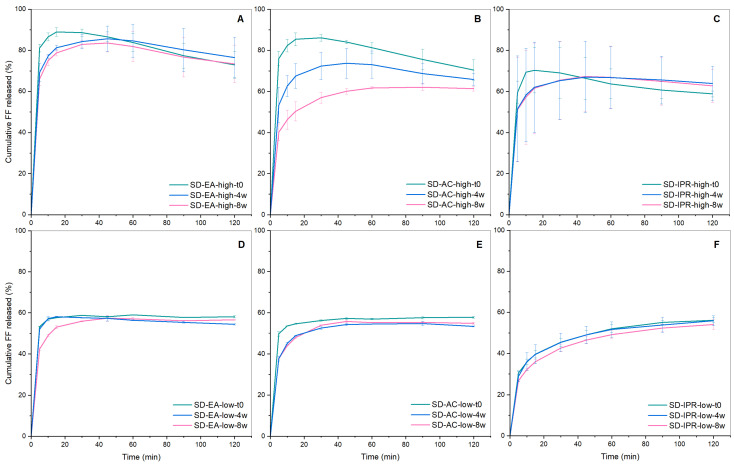
Drug release from SDs containing 30% FF prepared at different conditions (solvent, temperature) at time 0, week 4 and week 8 (storage at 40 °C, 75% RH); SD-EA-high (**A**), SD-AC-high (**B**), SD-IPR-high (**C**), SD-EA-low (**D**), SD-AC-low (**E**), SD-IPR-low (**F**).

The results of the DSC and XRPD studies after 8 weeks of storage at 40 °C and 75% RH for the samples prepared with different solvents and temperatures are shown in Figure 14 and Figure 15, respectively, while Figure 16 shows drug release after 4 and 8 weeks compared to the initial results (noted as t0). Although the vials were closed during storage, it can be assumed that water vapor was able to enter the container through the plastic lid or through the seal between the glass vial and the lid. In general, it is expected that the presence of water leads to the recrystallization of amorphous formulations, as water acts as a plasticizer that lowers T_g_ and increases molecular mobility. The elevated temperature also leads to higher molecular mobility and is associated with poor physical stability [49,50,51]. Therefore, it is not surprising that some samples show signs of crystallization, as evidenced by larger peaks in DSC and XRPD, as well as slower dissolution rates.

It can be seen from Figure 14 that there are endothermic events in each formulation except SD-EA-high, which means that this is the only formulation that shows no signs of FF crystallization and is the most stable according to the DSC measurements. On the other hand, the largest endothermic peaks are seen for the samples prepared with isopropanol, both at the boiling point temperature and at 40 °C. The larger peak for both samples (at 80 °C) is consistent with the melting of crystalline FF on the surface of Syloid particles, which indicates possible FF migration from the pores to the surface and its crystallization. Peaks are also seen at 60–65 °C, which indicates crystallization within the pores. Other samples (SD-AC-high, SD-AC-low, and SD-EA-low) also show endothermic events at 80 °C and/or at 60–65 °C, but these are significantly lower than for SD-IPR-high and SD-IPR-low.

The results obtained with XRPD show more or less the same picture; SD-EA-high, like SD-AC-high, shows no peaks. All other formulations show peaks, which means that FF is definitely partially crystalline in them. Furthermore, SDs prepared with isopropanol show the highest peaks, which is consistent with them showing the largest endothermic event based on DSC.

It should be emphasized that SD-EA-high appears to be completely amorphous after 8 weeks, according to both DSC and XRPD, while XRPD shows some crystalline peaks at the initial time point. It could be that XRPD has a higher sensitivity of both methods to detect crystalline material and that a small part of the analyzed sample at the initial time was crystalline by chance (note that the Bragg peaks are very small).

Looking at the results of the dissolution experiments (Figure 16), it can be seen that some results contradict the conclusions drawn from the DSC and XRPD results. For example, drug release from SD-AC-high is significantly decreased at weeks 4 and 8, compared to t0, although DSC and XRPD showed almost no change, which is very unusual and requires further investigation. On the other hand, SD-IPR-high and SD-IPR-low show no significant differences in drug release after storage or even indicate a greater drug release after 2 h (see curves SD-IPR-high at 4 and 8 weeks), although peaks characteristic of crystalline FF were clearly seen in DSC and XRPD, which should imply a lower dissolution rate and a lower extent of drug release. One possible explanation for the increased drug release after storage is that the adsorbed water molecules weaken the interactions between FF and the silica surface, which facilitates the release of FF from the surface upon contact with water [52]. Another possibility is that the presence of water vapor increases the hydroxylation of the silica surface and makes it more hydrophilic, which may lead to an increased release of the drug [21,52,53]. However, these explanations also require further evidence.

Another interesting observation is that all SDs, prepared at 40 °C, show almost no change in dissolution profile after storage for 4 or 8 weeks. A possible explanation for this is that a kind of equilibrium state was formed in these samples and that the adsorption has led to a more thermodynamically stable system than in other SDs. However, this is not in line with the DSC results, where signs of crystallization are visible. It should also be noted that the dissolution rate and extent of drug release were initially much lower for these samples than for those prepared at higher temperatures. A longer stability study could provide different results and lead to different conclusions. All in all, it is desirable that the SDs do not change structurally during the storage period and that their initial characteristics are retained.

**Figure 17:**
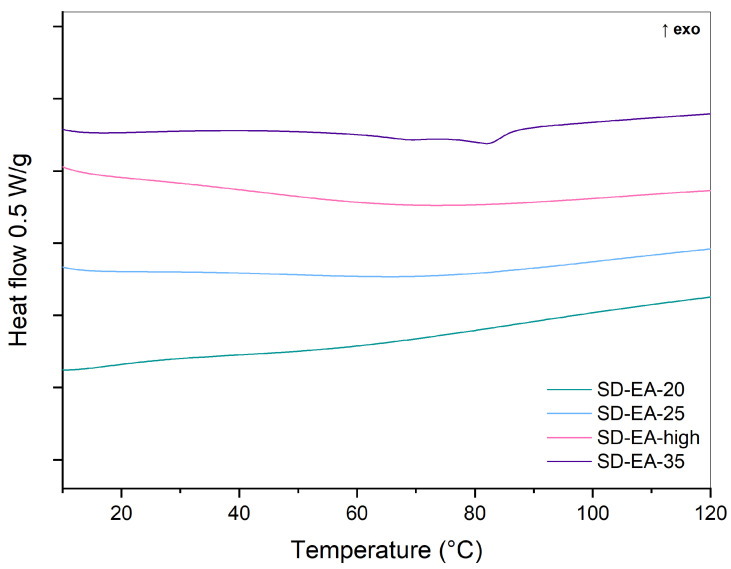
DSC curves of SDs prepared with different amounts of FF in ethyl acetate at boiling point temperature after 8 weeks of storage at 40 °C, 75% RH.

**Figure 18:**
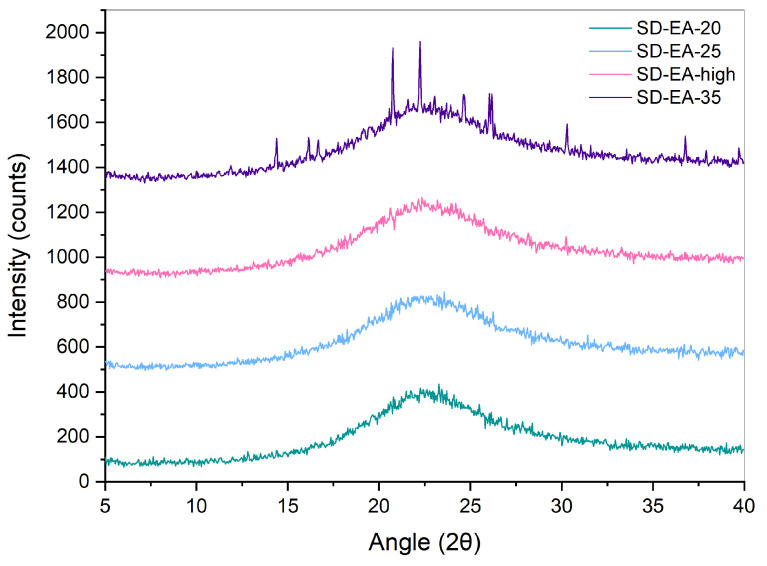
XRPD spectra of SDs prepared with different amounts of FF in ethyl acetate at boiling point temperature after 8 weeks of storage at 40 °C, 75% RH.

**Figure 19:**
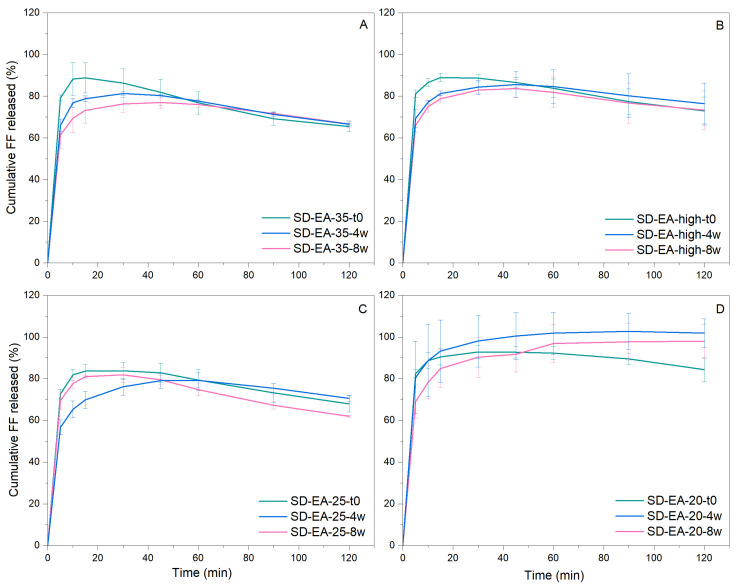
Drug release SDs prepared with different amounts of FF in ethyl acetate at boiling point temperature at time 0, week 4, and week 8 (storage at 40 °C, 75% RH); SD-EA-35 (**A**), SD-EA-high (**B**), SD-EA-25 (**C**), SD-EA-20 (**D**).

DSC and XRPD measurements as well as dissolution profiles of SDs with different FF contents prepared in ethyl acetate at boiling point temperature after storage are shown in Figure 17, Figure 18 and Figure 19. Samples with 20–30% FF show no endothermic events, while SD-EA-35 shows small peaks at 80 °C and 60–65 °C. This indicates that samples with 30% FF or less remain physically stable, while SD with 35% shows signs of recrystallization. This could be due to the possible presence of FF on the silica surface, where surface crystallization can occur more easily than in narrow pores with spatial constraints. The results from DSC were confirmed by XRPD, where distinct Bragg peaks are only seen in SD-EA-35, while other samples only show an amorphous halo.

The dissolution profiles of SD-EA-20, SD-EA-25, SD-EA-high, and SD-EA-35 (Figure 19) do not show very large changes between t0 and 8 weeks. SD-EA-35 shows the largest decrease in drug release, which is consistent with the DSC results. Other samples show deviations to either a higher or lower dissolution rate after 4 or 8 weeks of storage, but the differences in drug release profile at t0 are fairly small and mostly not significant.

## 4. Conclusions

The preparation of solid dispersions with mesoporous silica (Syloid) as a carrier was successfully used to improve the dissolution rate of the DCS Class IIb drug fenofibrate. It was confirmed that choosing the right preparation conditions in the solvent evaporation method is crucial to producing solid dispersions with the desired characteristics, i.e., an amorphous state that is stable during the storage period. While the solvent temperature seems to be very important, the choice of solvent seems to have a less significant influence. Further investigations are needed to confirm this hypothesis. Under suitable experimental conditions, we were able to prepare completely amorphous solid dispersions containing up to 30% fenofibrate, all of which exhibited fast drug release and supersaturation. Although amorphous fenofibrate is prone to physical instability, we have shown that its incorporation into mesoporous silica can be an excellent choice to maintain formulation characteristics; however, further testing is required to better understand the mechanisms that influence the physical stability of our products. The acquired results suggest that our approach has good potential to improve dissolution and ensure stability, even for the substances whose characteristics are the most difficult to improve.

## Figures and Tables

**Table 1:** The amount of components and conditions of preparation for the formulations.

Formulation	Type of Formulation	FF Mass (g)	Syloid Mass (g)	Theoretical FF Content (%)	Solvent	Solvent Evaporation Temperature (°C)
SD-EA-high	SD	2.4	5.6	30	Ethyl acetate	77 ± 2
SD-EA-low	SD	2.4	5.6	30	Ethyl acetate	40 ± 2
SD-AC-high	SD	2.4	5.6	30	Acetone	56 ± 2
SD-AC-low	SD	2.4	5.6	30	Acetone	40 ± 2
SD-IPR-high	SD	2.4	5.6	30	Isopropanol	82 ± 2
SD-IPR-low	SD	2.4	5.6	30	Isopropanol	40 ± 2
SD-EA-20	SD	1.6	6.4	20	Ethyl acetate	77 ± 2
SD-EA-25	SD	2.0	6.0	25	Ethyl acetate	77 ± 2
SD-EA-35	SD	2.8	5.2	35	Ethyl acetate	77 ± 2
PM-30	Physical mixture	2.4	5.6	30	-	-

**Table 2:** SSA, pore volume, and average pore diameter of SDs prepared with different amounts of FF in ethyl acetate at boiling point.

Sample	SSA (m^2^/g)	Pore Volume (mL/g)	Average Pore Diameter (nm)
Syloid	289	1.40	18.6
SD-EA-20	198	1.05	17.8
SD-EA-25	155	0.90	18.8
SD-EA-high	163	0.82	17.2
SD-EA-35	143	0.77	18.2

## Data Availability

Data are contained within the article.

## References

[1] Šoltys M., Kovačík P., Dammer O., Beránek J., Štěpánek F. (2019). Effect of Solvent Selection on Drug Loading and Amorphisation in Mesoporous Silica Particles. Int. J. Pharm..

[2] Azad M., Moreno J., Davé R. (2018). Stable and Fast-Dissolving Amorphous Drug Composites Preparation via Impregnation of Neusilin^®^ UFL2. J. Pharm. Sci..

[3] Baumgartner A., Planinšek O. (2021). Application of Commercially Available Mesoporous Silica for Drug Dissolution Enhancement in Oral Drug Delivery. Eur. J. Pharm. Sci..

[4] Chaudhari S.P., Gupte A. (2017). Mesoporous Silica as a Carrier for Amorphous Solid Dispersion. Br. J. Pharm. Res..

[5] Laitinen R., Löbmann K., Strachan C.J., Grohganz H., Rades T. (2013). Emerging Trends in the Stabilization of Amorphous Drugs. Int. J. Pharm..

[6] Price J.D. The Developability Classification System (DCS): Enabling an Optimized Approach for Formulation of Poorly Soluble Molecules. https://www.sigmaaldrich.com/deepweb/assets/sigmaaldrich/marketing/global/documents/363/277/wp8954en-solubility-mk.pdf.

[7] Sironi D., Rosenberg J., Bauer-Brandl A., Brandl M. (2017). Dynamic Dissolution-/Permeation-Testing of Nano- and Microparticle Formulations of Fenofibrate. Eur. J. Pharm. Sci..

[8] Yousaf A.M., Kim D.W., Oh Y.-K., Yong C.S., Kim J.O., Choi H.-G. (2015). Enhanced Oral Bioavailability of Fenofibrate Using Polymeric Nanoparticulated Systems: Physicochemical Characterization and in Vivo Investigation. Int. J. Nanomed..

[9] Zhang J., Wu C.-Y., Pan X., Wu C. (2017). On Identification of Critical Material Attributes for Compression Behaviour of Pharmaceutical Diluent Powders. Materials.

[10] Baumgartner A., Planinšek O. (2023). Effect of Process Parameters in High Shear Granulation on Characteristics of a Novel Co-Processed Mesoporous Silica Material. Eur. J. Pharm. Sci..

[11] Planinšek O., Kovačič B., Vrečer F. (2011). Carvedilol Dissolution Improvement by Preparation of Solid Dispersions with Porous Silica. Int. J. Pharm..

[12] Genina N., Hadi B., Löbmann K. (2018). Hot Melt Extrusion as Solvent-Free Technique for a Continuous Manufacturing of Drug-Loaded Mesoporous Silica. J. Pharm. Sci..

[13] Vialpando M., Albertini B., Passerini N., Bergers D., Rombaut P., Martens J.A., Van Den Mooter G. (2013). Agglomeration of Mesoporous Silica by Melt and Steam Granulation. Part I: A Comparison between Disordered and Ordered Mesoporous Silica. J. Pharm. Sci..

[14] Limnell T., Santos H.A., Mäkilä E., Heikkilä T., Salonen J., Murzin D.Y., Kumar N., Laaksonen T., Peltonen L., Hirvonen J. (2011). Drug Delivery Formulations of Ordered and Nonordered Mesoporous Silica: Comparison of Three Drug Loading Methods. J. Pharm. Sci..

[15] Sun W.-J., Aburub A., Sun C.C. (2018). A Mesoporous Silica Based Platform to Enable Tablet Formulations of Low Dose Drugs by Direct Compression. Int. J. Pharm..

[16] Escriche-Navarro B., Escudero A., Lucena Sánchez E., Sancenón F., García-Fernández A., Martínez-Máñez R. (2022). Mesoporous Silica Materials as an Emerging Tool for Cancer Immunotherapy. Adv. Sci..

[17] Xu Q., Yang Y., Lu J., Lin Y., Feng S., Luo X., Di D., Wang S., Zhao Q. (2022). Recent Trends of Mesoporous Silica-Based Nanoplatforms for Nanodynamic Therapies. Coord. Chem. Rev..

[18] Mazzotta E., De Santo M., Lombardo D., Leggio A., Pasqua L. (2022). Mesoporous Silicas in Materials Engineering: Nanodevices for Bionanotechnologies. Mater. Today Bio.

[19] Maleki A., Kettiger H., Schoubben A., Rosenholm J.M., Ambrogi V., Hamidi M. (2017). Mesoporous Silica Materials: From Physico-Chemical Properties to Enhanced Dissolution of Poorly Water-Soluble Drugs. J. Control. Release.

[20] Hussain T., Waters L.J., Parkes G.M.B., Shahzad Y. (2017). Microwave Processed Solid Dispersions for Enhanced Dissolution of Gemfibrozil Using Non-Ordered Mesoporous Silica. Colloids Surf. A Physicochem. Eng. Asp..

[21] Ahern R.J., Hanrahan J.P., Tobin J.M., Ryan K.B., Crean A.M. (2013). Comparison of Fenofibrate-Mesoporous Silica Drug-Loading Processes for Enhanced Drug Delivery. Eur. J. Pharm. Sci..

[22] Lai J., Lin W., Scholes P., Li M. (2017). Investigating the Effects of Loading Factors on the In Vitro Pharmaceutical Performance of Mesoporous Materials as Drug Carriers for Ibuprofen. Materials.

[23] Jia Z., Lin P., Xiang Y., Wang X., Wang J., Zhang X., Zhang Q. (2011). A Novel Nanomatrix System Consisted of Colloidal Silica and pH-Sensitive Polymethylacrylate Improves the Oral Bioavailability of Fenofibrate. Eur. J. Pharm. Biopharm..

[24] Khanfar M., Al-Nimry S. (2017). Stabilization and Amorphization of Lovastatin Using Different Types of Silica. AAPS PharmSciTech.

[25] Pardhi V., Chavan R.B., Thipparaboina R., Thatikonda S., Naidu V., Shastri N.R. (2017). Preparation, Characterization, and Cytotoxicity Studies of Niclosamide Loaded Mesoporous Drug Delivery Systems. Int. J. Pharm..

[26] Brunauer S., Emmett P.H., Teller E. (1938). Adsorption of Gases in Multimolecular Layers. J. Am. Chem. Soc..

[27] Lippens B.C., de Boer J.H. (1965). Studies on Pore Systems in Catalysts: V. The t Method. J. Catal..

[28] Barrett E.P., Joyner L.G., Halenda P.P. (1951). The Determination of Pore Volume and Area Distributions in Porous Substances. I. Computations from Nitrogen Isotherms. J. Am. Chem. Soc..

[29] Moore J.W., Flanner H.H. (1996). Mathematical Comparison of Dissolution Profiles. Pharm. Technol..

[30] Maulvi F.A., Dalwadi S.J., Thakkar V.T., Soni T.G., Gohel M.C., Gandhi T.R. (2011). Improvement of Dissolution Rate of Aceclofenac by Solid Dispersion Technique. Powder Technol..

[31] Waters L.J., Hussain T., Parkes G., Hanrahan J.P., Tobin J.M. (2013). Inclusion of Fenofibrate in a Series of Mesoporous Silicas Using Microwave Irradiation. Eur. J. Pharm. Biopharm..

[32] Heinz A., Gordon K.C., McGoverin C.M., Rades T., Strachan C.J. (2009). Understanding the Solid-State Forms of Fenofibrate--a Spectroscopic and Computational Study. Eur. J. Pharm. Biopharm..

[33] Matsumoto K., Nakai Y., Yonemochi E., Oguchi T., Yamamoto K. (1998). Effect of Pore Size on the Gaseous Adsorption of Ethenzamide on Porous Crystalline Cellulose and the Physicochemical Stability of Ethenzamide after Storage. Chem. Pharm. Bull..

[34] Mohanty S., Sahoo S., Patra S., Tripathy S. (2022). Design and Development of Fenofibrate Solid Dispersions for Solubility Enhancement. J. Pharm. Negat. Results.

[35] Wen T., Niu B., Wu Q., Zhou Y., Pan X., Quan G., Wu C. (2019). Fenofibrate Solid Dispersion Processed by Hot-Melt Extrusion: Elevated Bioavailability and Its Cell Transport Mechanism. Curr. Drug Deliv..

[36] Girgsdies F. (2015). Peak Profile Analysis in X-ray Powder Diffraction.

[37] Shi X., Shao Y., Sheng X. (2018). A New Polymorph of Fenofibrate Prepared by Polymer-Mediated Crystallization. J. Cryst. Growth.

[38] Figari G., Gonçalves J.L.M., Diogo H.P., Dionísio M., Farinha J.P., Viciosa M.T. (2023). Understanding Fenofibrate Release from Bare and Modified Mesoporous Silica Nanoparticles. Pharmaceutics.

[39] Jamadar S., Pore Y., Sayyad F. (2014). Formation of Amorphous Telmisartan Polymeric Microparticles for Improvement of Physicochemical Characteristics. Part. Sci. Technol..

[40] Wang L., Cui F.D., Sunada H. (2006). Preparation and Evaluation of Solid Dispersions of Nitrendipine Prepared with Fine Silica Particles Using the Melt-Mixing Method. Chem. Pharm. Bull..

[41] Watterson S., Hudson S., Svärd M., Rasmuson Å.C. (2014). Thermodynamics of Fenofibrate and Solubility in Pure Organic Solvents. Fluid Phase Equilibria.

[42] Sadeghi M., Rasmuson Å.C. (2020). Solubility of Salicylic Acid, Salicylamide, and Fenofibrate in Organic Solvents at Low Temperatures. J. Chem. Eng. Data.

[43] Illustrated Glossary of Organic Chemistry. https://www.chem.ucla.edu/~harding/IGOC/D/dielectric_constant.html.

[44] Hillerström A., Andersson M., Samuelsson J., van Stam J. (2014). Solvent Strategies for Loading and Release in Mesoporous Silica. Colloid Interface Sci. Commun..

[45] Physical Properties of Solvents. https://www.sigmaaldrich.com/deepweb/assets/sigmaaldrich/marketing/global/documents/614/456/labbasics_pg144.pdf.

[46] Kovačič B., Vrečer F., Planinšek O. (2011). Solid Dispersions of Carvedilol with Porous Silica. Chem. Pharm. Bull..

[47] Le T.-T., Elzhry Elyafi A.K., Mohammed A.R., Al-Khattawi A. (2019). Delivery of Poorly Soluble Drugs via Mesoporous Silica: Impact of Drug Overloading on Release and Thermal Profiles. Pharmaceutics.

[48] Dening T.J., Taylor L.S. (2018). Supersaturation Potential of Ordered Mesoporous Silica Delivery Systems. Part 1: Dissolution Performance and Drug Membrane Transport Rates. Mol. Pharm..

[49] Jelić D. (2021). Thermal Stability of Amorphous Solid Dispersions. Molecules.

[50] Siracusa V. (2012). Food Packaging Permeability Behaviour: A Report. Int. J. Polym. Sci..

[51] McCarthy C.A., Ahern R.J., Dontireddy R., Ryan K.B., Crean A.M. (2016). Mesoporous Silica Formulation Strategies for Drug Dissolution Enhancement: A Review. Expert Opin. Drug Deliv..

[52] Mellaerts R., Roeffaers M.B.J., Houthoofd K., Van Speybroeck M., De Cremer G., Jammaer J.A.G., Van den Mooter G., Augustijns P., Hofkens J., Martens J.A. (2011). Molecular Organization of Hydrophobic Molecules and Co-Adsorbed Water in SBA-15 Ordered Mesoporous Silica Material. Phys. Chem. Chem. Phys..

[53] Mellaerts R., Houthoofd K., Elen K., Chen H., Van Speybroeck M., Van Humbeeck J., Augustijns P., Mullens J., Van den Mooter G., Martens J.A. (2010). Aging Behavior of Pharmaceutical Formulations of Itraconazole on SBA-15 Ordered Mesoporous Silica Carrier Material. Microporous Mesoporous Mater..

